# Functional Studies on the IBD Susceptibility Gene IL23R Implicate Reduced Receptor Function in the Protective Genetic Variant R381Q

**DOI:** 10.1371/journal.pone.0025038

**Published:** 2011-10-12

**Authors:** Svetlana Pidasheva, Sara Trifari, Anne Phillips, Jason A. Hackney, Yan Ma, Ashley Smith, Sue J. Sohn, Hergen Spits, Randall D. Little, Timothy W. Behrens, Lee Honigberg, Nico Ghilardi, Hilary F. Clark

**Affiliations:** 1 Department of Bioinformatics and Computational Biology, Genentech Inc, South San Francisco, California, United States of America; 2 Department of Immunology, Genentech Inc, South San Francisco, California, United States of America; 3 ITGR Biomarker Discovery Group, Genentech Inc, South San Francisco, California, United States of America; 4 ITGR Early Development, Genentech Inc, South San Francisco, California, United States of America; 5 Department of Molecular Biology, Genentech Inc, South San Francisco, California, United States of America; 6 Genizon BioSciences, Inc., St. Laurent, Quebec, Canada; South Texas Veterans Health Care System, United States of America

## Abstract

Genome-wide association studies (GWAS) in several populations have demonstrated significant association of the *IL23R* gene with IBD (Crohn's disease (CD) and ulcerative colitis (UC)) and psoriasis, suggesting that perturbation of the IL-23 signaling pathway is relevant to the pathophysiology of these diseases. One particular variant, R381Q (rs11209026), confers strong protection against development of CD. We investigated the effects of this variant in primary T cells from healthy donors carrying IL23R^R381^ and IL23R^Q381^ haplotypes. Using a proprietary anti-IL23R antibody, ELISA, flow cytometry, phosphoflow and real-time RT-PCR methods, we examined IL23R expression and STAT3 phosphorylation and activation in response to IL-23. IL23R^Q381^ was associated with reduced STAT3 phosphorylation upon stimulation with IL-23 and decreased number of IL-23 responsive T-cells. We also observed slightly reduced levels of proinflammatory cytokine secretion in IL23R^Q381^ positive donors. Our study shows conclusively that IL23R^Q381^ is a loss-of-function allele, further strengthening the implication from GWAS results that the IL-23 pathway is pathogenic in human disease. This data provides an explanation for the protective role of R381Q in CD and may lead to the development of improved therapeutics for autoimmune disorders like CD.

## Introduction

The inflammatory bowel diseases (IBD), Crohn's disease (CD) and ulcerative colitis (UC), have long been recognized as having a genetic element to disease susceptibility [1], [2]. Following the discovery of NOD2 as a CD susceptibility gene, there have been over seventy susceptibility genes and loci implicated in IBD [3], especially with the advent of genome-wide association studies. These newly discovered genetic associations have shed light on the biological pathways involved in disease initiation and pathogenesis.

Several genes in the Th17 pathway have been linked with IBD susceptibility, including IL23R, TNFSF15, STAT3, IL12B, CCR6 and JAK2 [3]. Of all the Th17 pathway genes, the interleukin 23 receptor (*IL23R*) gene (GenBank accession: NM_144701, GeneID: 149233) on chromosome 1p31 confers the highest odds-ratio (OR) for disease development/lowest OR for disease protection [4], [5], [6], [7], [8], [9], [10], [11], [12], as well as being implicated in other chronic inflammatory diseases including psoriasis [13] and ankylosing spondylitis [14]. R381Q (rs11209026), the only coding IL23R variant identified by GWAS, confers an OR of 0.45 for Crohn's disease development (MAF 1.9% in CD, 7% in controls) [4], and an OR of 0.55 for UC development (MAF 3.7% in UC, 7% in controls) [15], implying a protective effect.

IL23R is most highly expressed on activated T cells, particularly of the Th17 subtype, natural killer (NK) cells and at lower levels on monocytes, macrophages and dendritic cells [16].

IL23R pairs with IL12RB1 to confer IL-23 responsiveness on cells expressing both receptor subunits [16], [17]. Upon activation by IL-23, IL23R signals through the JAK/STAT pathway. IL23R associates constitutively with JAK2 and, in a ligand-dependent manner, with STAT3, STAT1, STAT4 and STAT5 can also be activated by IL-23 [16]. On ligand binding, JAK2 phosphorylates IL23R at Tyr705, recruiting STAT3 to the receptor complex, where it is further phosphorylated by JAK2. Phosphorylated STAT3 homodimerizes and translocates to the nucleus where it triggers downstream expression of cytokines, including IL-17A, IL-17F, IL-22 and IL-21 in Th17 cells (reviewed in [18], [19]).

As well as functioning as a mediator of cytokine production in Th17 pathway cells, several studies also suggest that IL23R is a key player in the proliferation and survival of Th17 cells [17], [20], [21]. Studies in intestinal tissue have shown that IL-17F and IL-22 mRNA expression (induced via IL-23 signaling) are significantly increased in inflamed colonic lesions in CD compared to uninflamed biopsies, and that IL-22 is associated with a higher expression of inflammatory mediators [22], [23].

Because of its role in T cell biology and the compelling genetic evidence, we hypothesized that IL23R^Q381^ could potentially influence the response of the IL23R-expressing cells such as Th17 cells in the host. Indeed, a recent study by Di Meglio and colleagues [24] suggests that IL23R^Q381^ exerts its protective effects through attenuation of IL-23 induced Th17 function.

We investigated this possibility using primary T cells from healthy donors carrying IL23R^R381^ and IL23R^Q381^ haplotypes.

## Results

### Arginine 381 is absolutely conserved across different species

**Figure 1:**
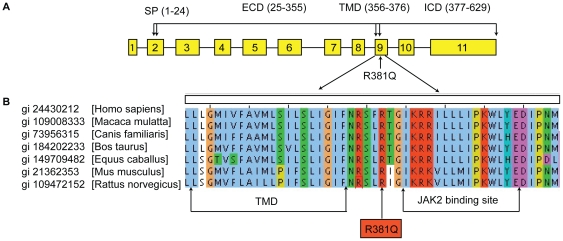
The arginine at position 381 of the IL23R is absolutely conserved across different species. (**A**) Map of the IL23R gene. (**B**) Sequence alignment of IL23R protein sequences from different species.

The R381Q polymorphism is located between the transmembrane domain and the putative JAK2 binding site in the cytoplasmic portion of IL23R protein, and is absolutely conserved across different species (Fig. 1 A, B). By virtue of its location, this polymorphism could potentially interfere with either surface localization of the IL23R [25] or signal transduction [26]; it is unlikely to interfere with ligand binding.

### Untransformed polyclonal IL23R^Q381^ positive T cell lines have decreased number of IL23R positive cells and reduced IL-23 responsiveness compared to IL23R^R381^ T cell lines

To study the effects of the R381Q polymorphism we used untransformed polyclonal T cell lines from donors carrying either IL23R^R381^ or IL23R^Q381^. IL23R positive cells were detected using proprietary monoclonal antibody against human IL23R (Fig. 1S). We genotyped 138 healthy donors and identified eighteen IL23R^Q381^ heterozygous individuals and one homozygous individual. The calculated allele frequency of 7.1% of R381Q is consistent with the published estimates (7.0% [4] 7.3% [27]; 6.0% [28]). We generated T cell lines from five IL23R^R381^, four IL23R^Q381^ heterozygous, and one IL23R^Q381^ homozygous donors. All donors were Caucasian of ages 25 to 65.

**Figure 2:**
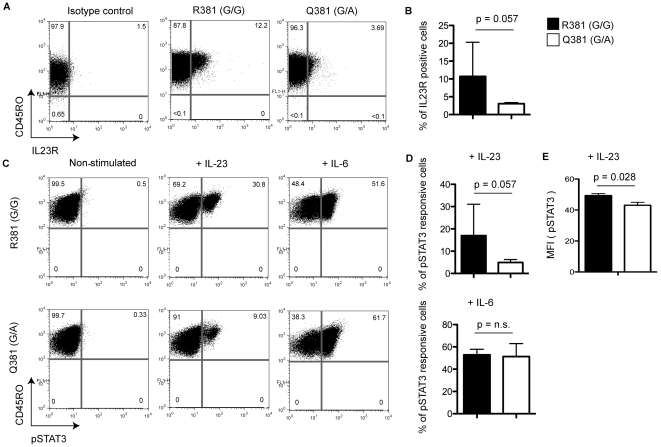
Untransformed polyclonal IL23R^Q381^ positive T cell lines have a decreased number of IL23R positive cells and reduced IL-23 responsiveness compared to IL23R^R381^ T cell lines. Flow cytometric analyses of T cell lines from IL23R^R381^ and IL23R^Q381^ donors is shown. Numbers in the quadrants indicate percent cells in that quadrant. (**A**) IL23R cell surface expression on non-permeabilized T cells of representative donors and (**B**) the mean percent (n = 4) of IL23R positive cells is shown. The error bars indicate standard deviation (SD). IL-23 and IL-6 induced STAT3 phosphorylation in (**C**) representative donors and (**D**) 4 donors per group. (**E**) The MFI pSTAT3 signal in the pSTAT3 positive population (n = 4). Data are representative examples of at least three independent experiments. The Mann-Whitney test was used to calculate the p value.

We observed no obvious differences in viability and proliferation rates between these T cell lines during *in vitro* expansion (Fig. S2A, B). However, flow cytometry analysis performed after six days of *in vitro* stimulation with feeder mixture (see Methods), revealed significantly diminished population of IL23R positive T cell from IL23R^Q381^ positive donors compared to their IL23R^R381^ counterparts (Fig. 2A, B). Consistently, when stimulated with IL-23 we observed fewer pSTAT3 positive cells in IL23R^Q381^ samples (Fig. 2C–D). In addition, the median fluorescence intensity (MFI) of IL-23 stimulated pSTAT3 positive cells was reduced (Fig. 2E), suggesting that not only was there a specific reduction of IL-23–responsive T-cells generated from IL23R^Q381^ positive individuals but also the strength of the IL-23 response for any given cell was decreased by the R381Q polymorphism. By comparison, IL-6-elicited STAT3 phosphorylation was unaffected by the IL23R genotype, demonstrating that IL23R^Q381^ positive cells are intrinsically capable of full STAT3 activation (Fig. 2C, D).

### Untransformed polyclonal IL23R^Q381^ positive T cell have decreased IL-23-induced pSTAT1 and pSTAT5 compared to IL23R^R381^ T cell lines

**Figure 3:**
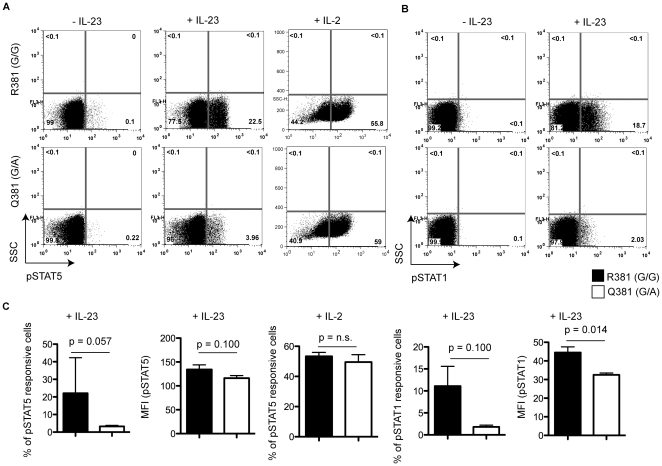
Untransformed polyclonal IL23R^Q381^ positive T cell have decreased IL-23 induced pSTAT1 and pSTAT5 compared to IL23R^R381^ T cell lines. (**A**) IL-23 and IL-2 induced pSTAT5 and (**B**) IL-23 induced pSTAT1 response in representative donors and (**C**) the mean of the percent positive from and MFI of pSTAT1 and pSTAT5 (n = 4). Data are representative examples of at least two independent experiments. The Mann-Whitney test was used to calculate the p value.

To further characterize this phenomenon, we examined IL-23-induced STAT1 and STAT5 phosphorylation. To reduce background levels of STAT5 phosphorylation elicited by endogenously produced IL-2, we added a neutralizing monoclonal anti-human IL-2 antibody to the cultures during IL-23 stimulation. We observed significantly decreased levels of both STAT5 (Fig. 3A, C) and STAT1 phosphorylation (Fig. 3B, C) in IL23R^Q381^ bearing T cells compared to the IL-23R^R381^ positive lines. The MFI of pSTAT5 positive cells was slightly decreased and pSTAT1 positive cells significantly decreased after IL-23 stimulation, in agreement with the defect observed in STAT3 phosphorylation. On the other hand, when stimulated with IL-2, pSTAT5 levels were equivalent between IL23R^R381^ and IL23R^Q381^ cell lines (Fig. 3A, C).

**Figure 4:**
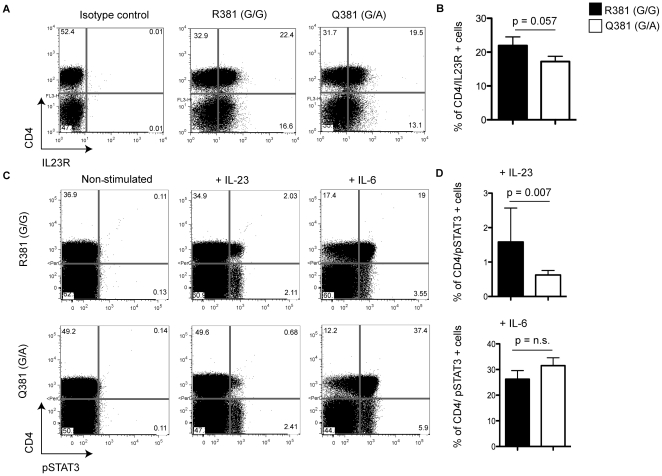
PBMCs from IL23R^Q381^ positive donors have decreased IL23R surface expression and reduced IL-23 responsiveness compared to IL23R^R381^. Flow cytometric analyses of PBMCs from IL23R^R381^ and IL23R^Q381^ donors is shown. Numbers in the quadrants indicate percent cells in each. (**A**) IL23R expression on non-permeabilized PBMCs stimulated with anti-CD3 and anti-CD28 antibodies for 72 hours of representative donors and (**B**) the mean of the percent positive from 4 donors per group is shown. The error bars indicate SD. IL-23 and IL-6 induced STAT3 phosphorylatin in whole blood from (**C**) representative donors and (**D**) 4 donors per group. Data are representative examples of at least three independent experiments. The Mann-Whitney test was used to calculate the p value.

In addition to T cell lines generated from IL23R^R381^ and IL23R^Q381^ donors, we analyzed freshly isolated peripheral blood mononuclear cells (PBMCs) from the same donors. IL23R expression is known to be activation dependent [29], and indeed we could not detect any IL23R expression on freshly isolated PBMC (data not shown). Therefore, we stimulated total PBMCs with agonist antibodies directed against CD3 and CD28 for 72 hours and then analyzed IL23R surface expression. After stimulation, we clearly observed IL23R expression on the IL23R^R381^ T cells, while the IL23R^Q381^ T cells had slightly, but not significantly decreased number of IL23R positive cells (Fig. 4A, B). We also observed decreased numbers of pSTAT3 positive IL23R^Q381^ CD4^+^ cells, compared to IL23R^R381^ CD4^+^ cells when we stimulated whole blood with IL-23. IL-6 stimulation resulted in similar numbers of pSTAT3 positive cells, regardless of IL23R genotype (Fig. 4C, D), confirming our data from T cell lines.

### Peripheral blood cytokine levels are slightly decreased in IL23R^Q381^ positive donors compared to IL23R^R381^

**Figure 5:**
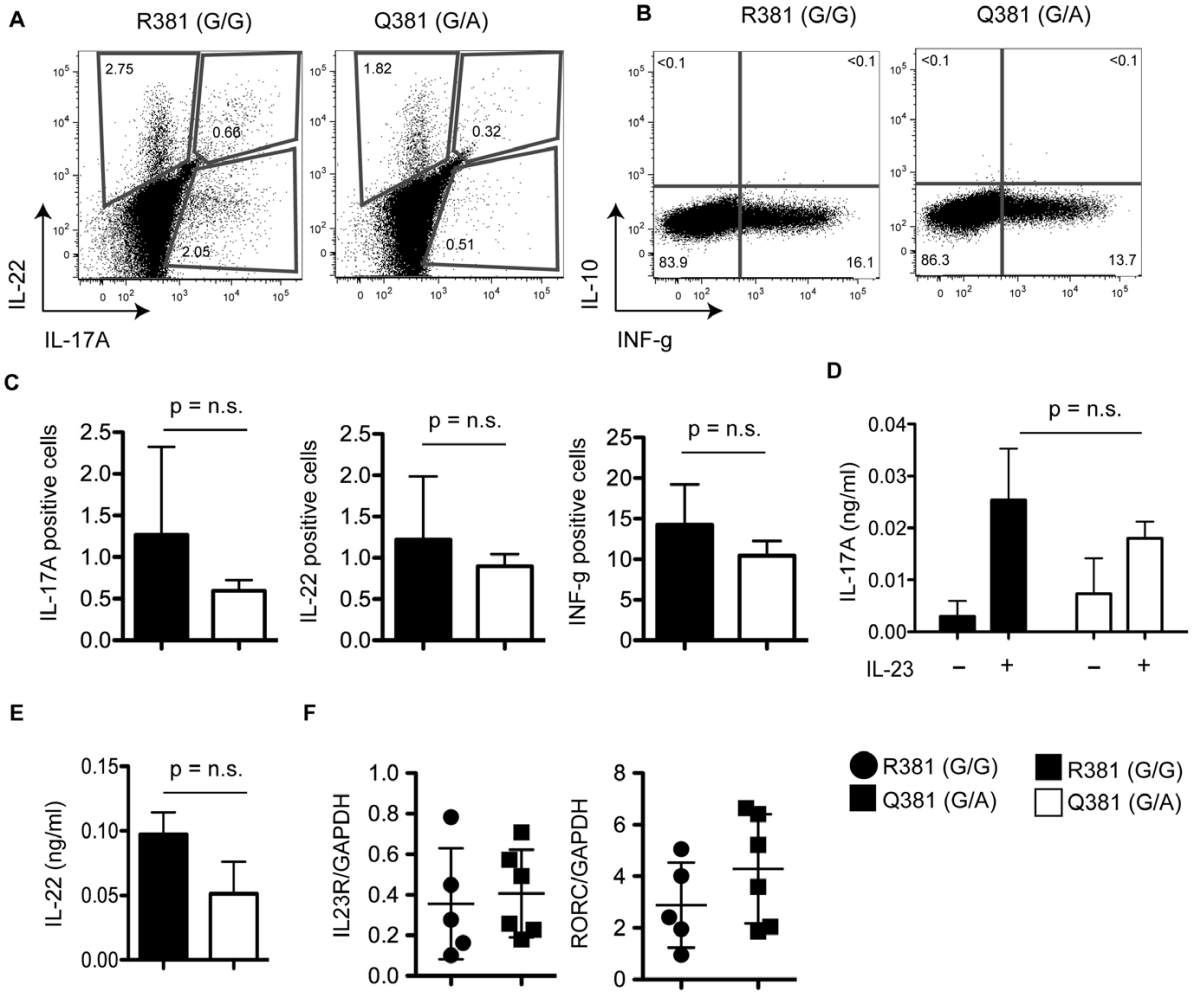
Peripheral blood cytokine levels are comparable in IL23R^Q381^ and IL23R^R381^ positive donors. ICS of cytokine production by T cell lines stimulated with anti-CD3/ CD28 dynabeads (**A**) representative donors for IL-22 and IL-17 and (**B**) IL-10 and INF-g (**C**) Frequency of cytokine positive cells (n = 4/per group). (**D**) Cytokine production by PBMCs stimulated with anti-CD3 and anti-CD28 antibodies+/−IL-23 and measured by ELISA (3 donors per group) (**E**) Serum IL-22 levels in IL23R^Q381^ and IL23R^R381^ positive donors (5 donors per group) measured by ELISA. (**F**) Real-time PCR analysis for IL23R and RORC mRNA expression, presented (in arbitrary units (AU)) relative to GAPDH expression (used as an internal “housekeeping” control). mRNA collected from PBMCs of IL23R^R381^ (5 donors) and IL23R^Q381^ (6 donors). Each symbol represents a donor. Data are representative examples of at least two independent experiments. The Mann-Whitney test was used to calculate the p value.

To investigate the functional impact of the IL23R^R381Q^ in terms of cytokine secretion, we used stimulated T cell lines from IL23R^R381^ and IL23R^Q381^ donors to measure the production of cytokines by intracellular staining (Fig. 5A–C). We observed slightly, but not significantly decreased levels of IL-17A, IL-22 and IFN-g in IL23R^Q381^ positive donors. Similar results were observed when fresh PBMCs from IL23R^R381^ and IL23R^Q381^ positive donors were stimulated (Fig. S3A–C). Interestingly, a recent study [30] using primary T cells from IL23R^R381^ (n = 27) and IL23R^Q381^ (n = 13) positive donors showed that IL23R^Q381^ donors have decreased IL-23–dependent IL-17 and IL-22 production. Serum IL-22 levels were determined using enzyme-linked immunosorbent assay (ELISA), and we again observed a slight, but not significant, trend toward decreased IL-22 production in IL23R^Q381^ donors compared to IL23R^R381^ donors, in agreement with previously published data [31]. We also extracted RNA from fresh PBMCs and analyzed IL23R and RORC (a Th17 marker) mRNA expression by real-time PCR. We observed heterogeneity among donors, but no significant differences between IL23R^R381^ and IL23R^Q381^ positive donor groups (Fig. 5F), which could be attributed either to the low number of donors or the possibility that this variant is has no effect on mRNA processing.

## Discussion

In the past several years, GWAS have lead to major breakthroughs in our understanding of the genetics of CD. However, there have been relatively few published studies functionally validating disease-associated variants so far [24], [30], [32], [33]. We chose several relevant *in vitro* systems to demonstrate conclusively that the R381Q variant results in decreased population of IL-23 responsive cells, as well as an attenuation of signaling via the IL23R, manifesting in diminished IL-23 dependent activation of all STATs known to be associated with IL-23 signaling. Supporting our results, a recent study [24] using *in vitro* polarized Th17 cell from IL23R^R381^ (n = 22) and IL23R^Q381^ (n = 19) positive donors demonstrated that the cells from L23R^Q381^ positive donors had impaired IL-23-mediated IL-17A production and STAT3 phosphorylation.

Several mechanisms might contribute to this observation, including reduced capacity of IL23R^Q381^ to activate STAT proteins due to impaired association of JAK2 proteins with the cytoplasmic tail of the receptor. Arginine 381 is located near the JAK2 putative binding site (Fig. 1). However, in order to determine whether R381Q is truly a causative variant and not a tagging single nucleotide polymorphism (SNP), which tags a protective IL23R haplotype associated with decrease population of IL23R positive/responsive cells, further studies such as generation of a R381Q knock-in mouse model would have to be conducted.

One of the major advantages of our study was the availability of healthy donors willing to donate blood repeatedly for our primary T cells studies. It should be noted that, while we used T cells in our analysis, the protective effect of IL23R^Q381^ is likely to be co-mediated by other IL-23 responsive cell types (e.g. dendritic cells [16]) *in vivo*). We observed decreases in cytokine levels in IL23R^Q381^ positive donors compared to IL23R^R381^ donors in peripheral blood. However, the difference was not significant, potentially because of the small number of available samples.

For future studies it will be important to analyze a much larger cohort of donors. In addition, it will be interesting to conduct deep sequencing studies to identify additional genetic variants harbored in IL23R. Indeed, recent study by Monozawa and colleagues [34] reported a number of low frequency IL23R variants identified by resequencing of positional candidates.

It is important to note that despite the R381 risk variant having an allelic frequency of greater than 90% in Caucasian populations, the prevalence of CD in North American populations is only approximately 0.2% [35] [36]. This underlies the polygenic nature of CD susceptibility and the importance of other genes (e.g. *NOD2*, *ATG16L1*) as well as environmental influences (e.g. smoking, diet and possibly bacteria) on the risk of CD development.

IL23R is a key player in the proliferation and survival of Th17 cells, which are critical for host defense against bacterial, fungal and viral infections at mucosal surfaces [37]. Negative evolutionary selection to insure an appropriate defense response may explain why only a small percentage of the population have this variant.

**Figure 6:**
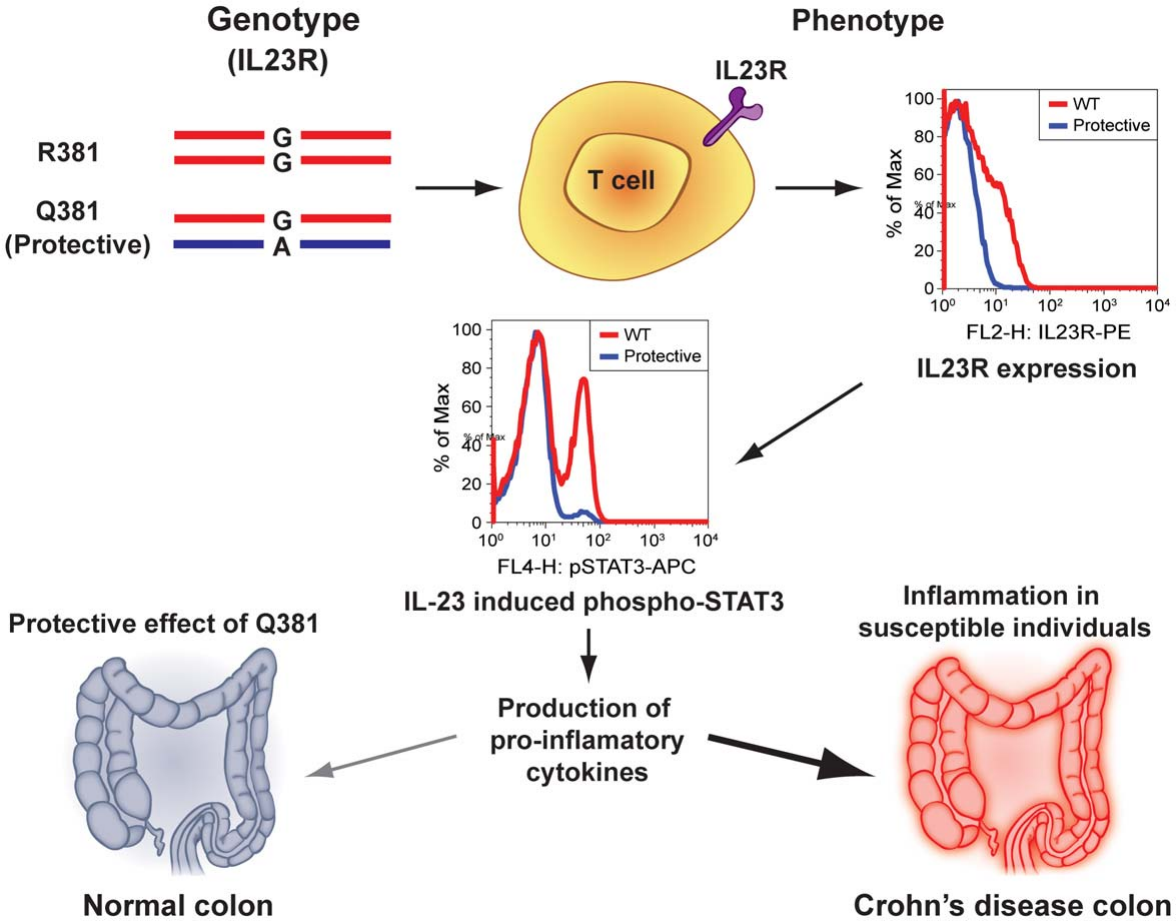
A summary of the results obtained in this study using genotype-selectable normal donors is depicted. IL23R^Q381^ donors had a significant decrease in IL23R positive T cells, leading to the decreased IL-23 induced STAT3 phosphorylation. Decreased STAT3 signaling in T cells like TH17 might modulate the extent and duration of the response in the host, leading to decreased secretion of proinflamatory cytokines such as IL-17 and IL-22 in the gut, explaining the protective effect of R381Q variant in CD and other autoimmune disorders.

In summary, our data indicate that IL23R^Q381^ is a hypomorphic IL23R allele that results in a decreased population of IL-23 responsive cells, leading to diminished IL-23-induced STAT3 phosphorylation. This provides an explanation for the protective role of R381Q in CD and other autoimmune disorders (Fig. 6) and further supports the hypothesis that blocking the IL-23 pathway may lead to improved therapeutics for autoimmune disorders like CD [38] and psoriasis [39].

## Materials and Methods

### Antibodies and ELISA

Information for all antibodies used in this study is summarized in Table S1. A monoclonal antibody against human IL23R (clone 20G3.4) was generated by immunizing mice with a hIL23R-Ig fusion protein (Fig. 1S).

Biotinylated and FITC-conjugated versions of this antibody were used in this study. Serum IL-22 levels of human genotype specific healthy donors were measured using the IL-22 ELISA MAX Set Deluxe kit (BioLegend, Inc) according to the manufacturer's instructions. Human IL-17A levels were measured in supernatants from PBMCs isolated from genotype specific healthy donors and stimulated with anti-CD3 (2.5 ug/ml) and anti-CD28 (1 ug/ml) antibodies+/−IL-23 (5 ng/ml) for 2 days. Supernatants were harvested and analyzed using hIL-17A ELISA (eBioscience) according to the manufacturer's instructions.

### IL23R construct

Gateway recombination cloning technology (Invitrogen) was used to create all constructs. Full-length wild-type *IL23R* coding sequence was PCR amplified using following primers: forward 5′ GG**ACAAGTTTGTACAAAAAAGCAGGCT**TCACC*ATG* *AATCAGGTCACTATT* - 3′ (bold - attB1 site; underlined - kozak; italics - Il23R nucleotides 1–18). Reverse 5′ – GGGG**ACCACTTTGTACAAGAAAGCTGGGTC** CTA *CTTTTCCAAGAGTGA* - 3′ (bold - attB1 site; underlined - stop; italics - Il23R nucleotides 1872–1886). PCR product was cloned into the pDONR221 donor vector (Invitrogen, Carlsbad, CA). Construct encoding IL23R^R381^ was subsequently transferred into a gateway adapted pMSCVpuro plasmid using LR Clonase, and final destination construct was sequence verified.

### BaF3 cell culture and transduction

BaF3 cells [40] were maintained in RPMI supplemented with 10% bovine calf serum, L-Glutamine and Penicillin-Streptomycin (Invitrogen, Carlsbad, CA). Conditioned medium from WEHI-3B cells was used as a source of IL-3 and added to the culture at 2% final concentration. A pMSCV- based plasmid encoding hIL-12RB1 was introduced by electroporation, and positive single cell clones were identified and sorted by FACS into individual wells of 96-well plates. Human IL23R^R381^ cDNA, cloned in the pMSCVpuro retroviral expression vector, was introduced into the same hIL-12RB1 containing BaF3 subclones by standard retroviral transduction. 293T cells in combination with the retroviral packaging vector pCL-Eco (Imgenex) were used as a packaging system. Twenty-four hours after transduction, BaF3 cells were put in 1 µg/ml puromycin (Clontech) medium to select transduced cells. Relative IL23R and IL12RB1 mRNA abundances were verified by qPCR.

To detect IL23R cell surface expression BaF3 cells were stained with biotinylated IL23R antibody 20G3.4 in combination with streptavidin-PE (eBioscience). Isotype control antibodies were used as negative controls (BD biosciences).

### Genotyping

Blood samples were obtained from healthy donors after written informed consent was provided. Ethical approval for the use of this material was obtained from the Western Institutional Review Board. Genomic DNA was isolated from 138 healthy donors, and the R381Q variant (rs11209026) was genotyped using Applied Biosystems TaqMan SNP genotyping assay (assay ID: C_1272298_10). Eighteen donors were identified as being IL23R^Q381^ heterozygous (GA) and 1 homozygous (AA).

### T cell lines

T cell lines were generated as previously described [41], [42], [43]. Briefly, CD4^+^ T cells were sorted from whole blood of IL23R^R381^ and IL23R^Q381^ positive donors using a human whole blood CD4 selection kit (RoboSep; StemCell Technologies) according to the manufacturer's instructions (purity of the CD4+ cells after enrichment was >95%). Cells were seeded at 5×10^5^ cells/ml and stimulated with a feeder mixture containing 1×10^6^/ml irradiated (6,000 rad) allogenic PBMC and 1×10^5^/ml irradiated (10,000 rad) JY cells, 1 µg/ml phytohemagglutinin (Sigma), and 200 IU/ml recombinant human IL-2 (Roche). Cells were cultured in Yssel's medium (Gemini Bio-Products) supplemented with 1% human serum. T cells were restimulated with the feeder mixture every 2 weeks. Cell surface expression of IL23R was analyzed by flow cytometry 6 days after stimulation.

### T cell culture

PBMCs were isolated from whole blood of IL23R^R381^ and IL23R^Q381^ positive donors using UNI-SEP (U-10, Accurate Chemical) pre-filled tubes containing a solution of 5.6% polysucrose and 9.6% sodium metrizoate (density 1.077 g/ml, osmolality 280 mOsm). Cells were stimulated with anti-CD3 (2.5 ug/ml) and anti-CD28 (1 ug/ml) antibodies for 72 hours. IL23R cell surface cell surface expression was analyzed by flow cytometry using biotinylated IL23R antibody in combination with streptavidin-PE (eBioscience).

### Cell viability Assay

T cells were plated in 96-well plates in triplicates at 5×10^5^ cells per well. IL-23 was added to a final concentration of 100 nM and a 1∶4 dilution series was established. After 72 h of stimulation cells were analyzed by the CellTiter-Glow Luminescent Cell Viability Assay according to the instructions of the manufacturer (Promega Corp). All experiments were performed in triplicate wells.

### Proliferation Assay

T cells were plated in 96-well plates in triplicates at 5×10^5^ cells per well. IL-23 was added to a final concentration of 100 nM and a 1∶2 dilution series was established. After 72 h of stimulation cells were analyzed by a 16-h pulse with 1 µCi/well [^3^H] thymidine (Amersham Biosciences) followed by harvesting using a 96-well plate harvester and counting by liquid scintillation. All experiments were performed in triplicate wells.

### Flow cytometry

All data were collected on FacsCalibur and LSR II instruments (BD Biosciences) and analyzed using FlowJo software (Tree Star). A summary of all antibodies used in this study is provided in Table S1, and antibodies were used according to the instructions of the manufacturer unless otherwise indicated.

### IL23R cell surface expression

T cells were stained with biotinylated IL23R antibody 20G3.4 in combination with streptavidin-PE (eBioscience). Isotype control antibodies were used as negative controls (BD biosciences).

### STAT phosphorylation

Flow cytometric analyses of STAT phosphorylation were performed as previously described [43], [44]. Briefly, T cells were starved overnight in Yssel's medium supplemented with monoclonal anti-human IL-2 antibody to neutralize IL-2. Cells were stimulated with 10 ng/ml of rhIL-23 or 10 ng/ml of rhIL-6 or 1000 IU/ml of IL-2 and incubated at 37°C for 15 min. Cells were fixed, permeabilized and stained with phospho-STAT3, phospho-STAT1 and phospho-STAT5 specific antibodies to detect activated STAT3, STAT1 and STAT5, respectively.

Whole blood was stimulated with 10 ng/ml of IL-23 or 10 ng/ml of rhIL-6 and incubated at 37°C for 15 min, fixed and lysed to halt signaling and lyse red blood cells. Cells were permeabilized and stained with phospho-STAT3 specific antibodies to detect activated STAT3.

### Intracellular cytokine staining (ICS)

Intracellular staining was done as previously described [45]. Briefly, T cells were stimulated either with Dynabeads CD3/CD28 T Cell Expander (Invitrogen) (bead∶cell ratio = 1∶10) or with 10 ng/ml PMA plus 500 ng/ml ionomycin (Sigma). After 3 hrs, BD Golgi Plug with brefeldin A (BD Biosciences) was added to block secretion. After an additional 3 hrs, T cells were stained with green live/dead dye (Invitrogen). Cells were fixed in 3% paraformaldehyde and permeabilized with Perm/Wash buffer (BD Biosciences). Permeabilized T cells were stained with anti-hIFN-g, anti-hIL-17, anti-hIL-10, anti-hIL-22 or isotype control antibodies (BD Biosciences).

### Real-time quantitative RT-PCR

Total RNA was extracted from freshly isolated PBMCs of IL23R^R381^ and IL23R^Q381^ positive donors using RNeasy Micro kit (Qiagen). High-Capacity cDNA Archive kit (Applied Biosystems) was used for cDNA synthesis. Transcripts were quantified by real-time quantitative PCR on an ABI PRISM 7900 sequence detector (Applied Biosystems) with Applied Biosystems predesigned TaqMan Gene Expression Assays. The following probes were used: RORC (Hs01076112) and IL23R (Hs00332759). For each sample, mRNA abundance was normalized to the amount of GAPDH or RPL19 transcripts.

### Sequence alignment by CLUSTAL W

IL23R protein sequences from all the species available in Genbank were aligned with Clustal W in FASTA format (http://www.ebi.ac.uk/clustalw/).

### Statistical analyses

Statistical analyses were performed by the Mann-Whitney test using R and Graph Pad Prism software was used to calculate standard deviation. P<0.05 considered statistically significant.

## Supporting Information

Figure S1 The specificity of anti-IL23R antibody is demonstrated using BaF3 cells retrovirally transduced with IL23R^R381^. Representative analysis by flow cytometry show IL23R surface expression on non-permeabilized transduced BaF3 cells. Isotype control is indicated by gray shading, non-transformed BaF3 cells by a red line and IL23R^R381^ by a blue line.
(TIF)

Figure S2 Untransformed polyclonal IL23R^Q381^ positive T cell have comparable cell viability and proliferation rates to IL23R^R381^ cells. The mean percent (n = 4) of representative donors (**A**) cell viability and (**B**) proliferation rates after stimulation with IL-23 for 72 h. Data are representative examples of at least three independent experiments.
(TIF)

Figure S3 Cytokine levels are comparable in IL23R^Q381^ and IL23R^R381^ positive donors. ICS of cytokine production by PBMCs stimulated with anti-CD3/ CD28 dynabeads (**A**) representative donors for IL-22 and IL-17 and (**B**) IL-10 and INF-g (**C**) four donors per group is shown. Data are representative examples of at least three independent experiments. The Mann-Whitney test was used to calculate the p value.
(TIF)

Table S1 List of anti-human antibodies used in this study.
(DOCX)
