# Supplementary figures and images for: Functional Studies on the IBD Susceptibility Gene IL23R Implicate Reduced Receptor Function in the Protective Genetic Variant R381Q
Source: PLoS One. 2011 Oct 12;6(10):e25038. doi: 10.1371/journal.pone.0025038 (PMC3192060; doi:10.1371/journal.pone.0025038)

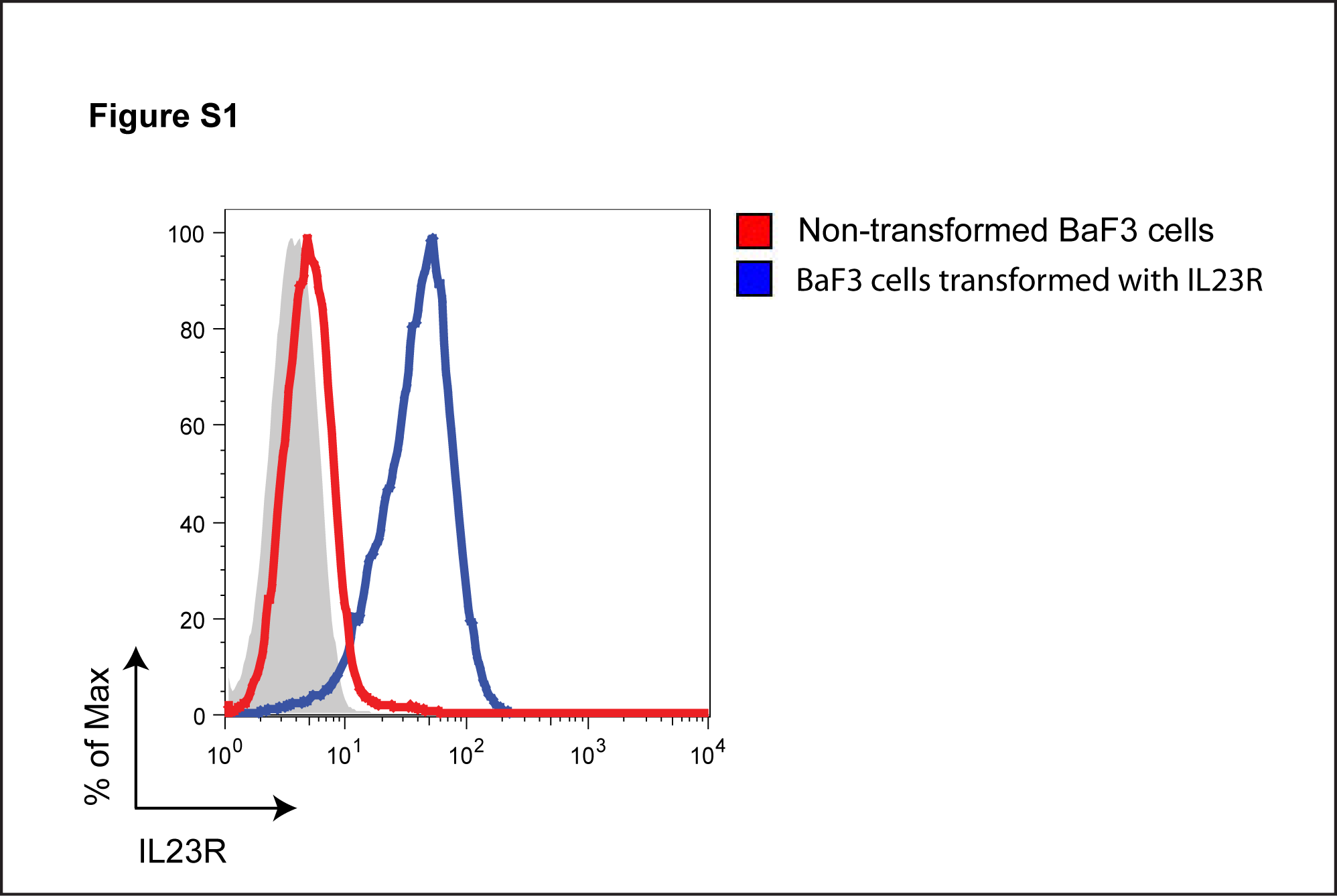

Supplement: Figure S1 — The specificity of anti-IL23R antibody is demonstrated using BaF3 cells retrovirally transduced with IL23RR381. Representative analysis by flow cytometry show IL23R surface expression on non-permeabilized transduced BaF3 cells. Isotype control is indicated by gray shading, non-transformed BaF3 cells by a red line and IL23RR381 by a blue line. (TIF) [file pone.0025038.s001.tif]

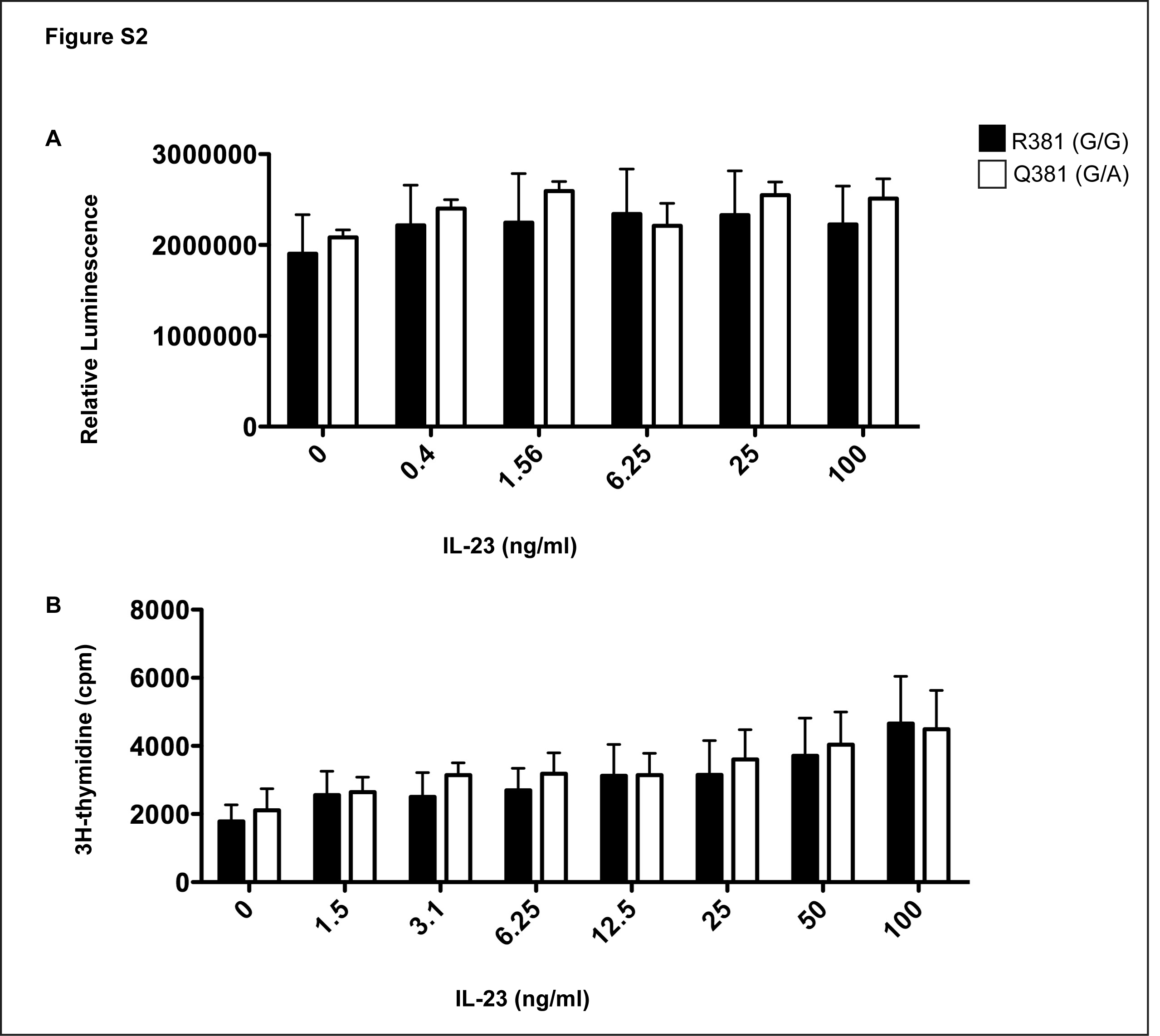

Supplement: Figure S2 — Untransformed polyclonal IL23RQ381 positive T cell have comparable cell viability and proliferation rates to IL23RR381 cells. The mean percent (n = 4) of representative donors (A) cell viability and (B) proliferation rates after stimulation with IL-23 for 72 h. Data are representative examples of at least three independent experiments. (TIF) [file pone.0025038.s002.tif]

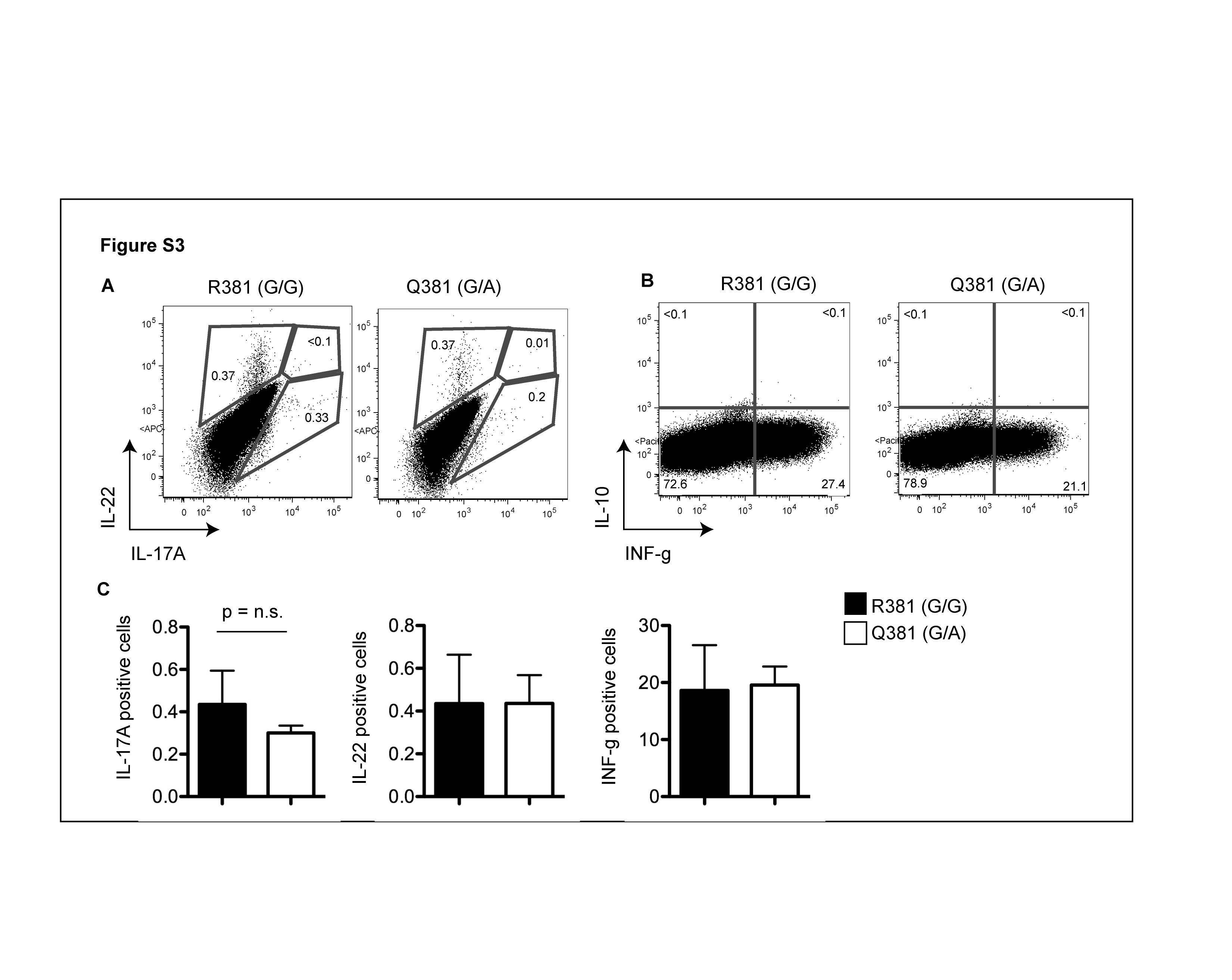

Supplement: Figure S3 — Cytokine levels are comparable in IL23RQ381 and IL23RR381 positive donors. ICS of cytokine production by PBMCs stimulated with anti-CD3/ CD28 dynabeads (A) representative donors for IL-22 and IL-17 and (B) IL-10 and INF-g (C) four donors per group is shown. Data are representative examples of at least three independent experiments. The Mann-Whitney test was used to calculate the p value. (TIF) [file pone.0025038.s003.tif]
