# Supplementary material for: Functional Studies on the IBD Susceptibility Gene IL23R Implicate Reduced Receptor Function in the Protective Genetic Variant R381Q
Source: PLoS One. 2011 Oct 12;6(10):e25038. doi: 10.1371/journal.pone.0025038 (PMC3192060; doi:10.1371/journal.pone.0025038)
Supplement: Table S1 — List of anti-human antibodies used in this study. (DOCX) [file pone.0025038.s004.docx]

**Table S1.**

| **Antigen** | **Manufacturer** | **Clone** | **Format** | **Application** |
| --- | --- | --- | --- | --- |
| IL23R | Genentech Inc | 20G3.4 | biotin | Flow cytometry |
| IL23R | Genentech Inc | 20G3.4 | FITC | Flow cytometry |
| CCR6 | R&D Systems | FAB195A | APC | Flow cytometry |
| CXCR3 | BD Bioscience  Pharmingen | 1C6/CXCR3 | PE-CY5 | Flow cytometry |
| CD45RA | BD Bioscience  Pharmingen | HI100 | FITC | Flow cytometry |
| CD25 | BD Bioscience  Pharmingen | M-A251 | FITC | Flow cytometry |
| CCR4 | BD Bioscience  Pharmingen | 1G1 | PECY7 | Flow cytometry |
| CD45RO | BD Bioscience  Pharmingen | UCHL1 | FITC | Flow cytometry |
| CD4 | BD Bioscience  Pharmingen | RPA-T4 | APC CY7 | Flow cytometry |
| hIL-2 | R&D Systems | 5334 | pure | Neutralization |
| IL-22 | Genentech Inc | 3F11 | Alexa-Fluor 647 | Flow cytometry |
| IFN-g | BD Bioscience | B27 | PECY7 | Flow cytometry |
| IL-17A | eBioscience | eBio64DEC17 | PE | Flow cytometry |
| IL-10 | eBioscience | JES3-9D7 | Pacific Blue | Flow cytometry |
| pSTAT1 (pY701) | BD Bioscience  Pharmingen | 4a | Alexa-Fluor 488 | Flow cytometry |
| pSTAT3 (pY705) | BD Bioscience  Pharmingen | 4/P-STAT3 | Alexa-Fluor 647 | Flow cytometry |
| pSTAT5 (pY694) | BD Bioscience  Pharmingen | 47 | PE | Flow cytometry |
| CD3 | BD Bioscience  Pharmingen | HIT3a | APC | Flow cytometry |
| CD8 | BD Bioscience  Pharmingen | RPA-T8 | APC | Flow cytometry |
| IL12Rβ1 | R&D Systems | FAB839P | PE | Flow cytometry |
| CD28 | BD Bioscience  Pharmingen | CD28.2 | pure | Stimulation |
| CD3 | eBioscience | OKT3 | pure | Stimulation |
| CD28 | BD Bioscience  Pharmingen | CD28.2 | pure | Stimulation |
| CD3 | eBioscience | OKT3 | pure | Stimulation |
| CD161 | eBioscience | DX12 | PE-Cy5 | Flow cytometry |
| IL1R1 | R&D Systems | FAB269P | PE | Flow cytometry |
